# Antibiotics in honey: a comprehensive review on occurrence and analytical methodologies.

**DOI:** 10.12688/openreseurope.17664.2

**Published:** 2024-11-08

**Authors:** Helena Rodrigues, Marta Leite, Beatriz Oliveira, Andreia Freitas

**Affiliations:** 1 University of Porto, Faculty of Pharmacy, Rua de Jorge Viterbo Ferreira 228, Porto, 4050-313, Portugal; 2 National Institute for Agricultural and Veterinary Research, Rua dos Lagidos, Lugar da Madalena, Vila do Conde, 4485-655, Portugal; 3 REQUIMTE/LAQV, R. D. Manuel II, Apartado 55142, Porto, Portugal, Porto, Portugal

**Keywords:** Antibiotic Residues; Honey; Occurrence; Extraction; liquid chromatography; mass spectrometry.

## Abstract

Honey is a food of great nutritional importance and has always been used for human consumption. The production of honey and other beekeeping products depends on the proper functioning of this extremely important sector, as it has a direct impact on other sectors such as agriculture. The decline in bee colony numbers has been linked, among other factors, to bacterial diseases affecting bees, including American and European foulbrood, and *Nosema* spp. disease. In this matter, prophylactic or therapeutic use of veterinary drugs in apiculture is common but can lead to their accumulation in bees and in honey. Consumption of contaminated honey can have adverse effects such as allergic or hypersensitivity reactions, carcinogenicity, reproductive effects, and teratogenicity. Commission Regulation (EU) N ^⍛^ 37/2010 sets MRLs for antibiotics in various foods, but these limits are not set for api-products. The lack of harmonized rules has led some countries to set recommended concentrations and minimum performance limits. Nonetheless, to achieve this goal, development of accurate and precise analytical methodologies is crucial. In recent years, the analysis of antibiotics in honey has led to the development of methods in an extensive range of families, including aminoglycosides, amphenicols, lincosamides, macrolides, nitroimidazoles, quinolones, sulfonamides, tetracyclines and nitrofurans. This review work entails an in-depth exploration of occurrence studies, extraction methodologies, and analytical techniques for the determination of antibiotics in apiculture products. It was found that the most used extraction methods include solid-phase extraction, dispersed solid or liquid phase extraction and QuEChERS. Due to the complexity of the honey matrix, samples are often diluted or acidified using McIlvaine buffer, H _2_O, MeOH, acidified ACN and TCA solution. This is usually followed by a purification step using SPE cartridges or PSA. Golden analytical methodologies include high-performance liquid chromatography coupled to a triple quadrupole mass spectrometer (MS/MS) with Orbitrap or Q-ToF detectors.

## Introduction

Honey is one of the oldest foods consumed and was used as a sweetener around the world for many years, until the introduction of sugar cane. Honey's nutritional and medicinal properties make it very appealing to consumers, who are increasingly adopting more organic diets and opting for less processed foods. It is commonly consumed in its natural form but also as an ingredient in recipes and food preparations ( Demirhan & Demirhan, 2022).

As according to Council Directive 2014/63/EU, honey is defined as “natural sweet substance produced by *Apis mellifera* bees from the nectar of plants or from secretions of living parts of plants or excretions of plant-sucking insects on the living parts of plants, which the bees collect, transform by combining with specific substances of their own, deposit, dehydrate, store and leave in honeycombs to ripen and mature.” ( European Commission, 2014). In this regulatory document, a distinction between two types of honey is also described based on their origin: blossom honey or nectar honey (obtained from the nectar of plants) and honeydew honey (obtained mainly from excretions of plant sucking insects ( *Hemiptera*) on the living part of plants or secretions of living parts of plants). Furthermore, this food can be classified based on the production method and/or presentation, namely as comb honey, chunk honey or cut comb in honey, drained honey, extracted honey, pressed honey, or filtered honey.

In recent years, the world honey production has increased steadily, reaching around 1,862,000 tonnes ( Gabinete de Planeamento, Políticas e Administração Geral, 2020). China is the world's largest producer, with an overall production of around 543,000 tonnes in 2017, followed by the European Union (230,000 tonnes), and Türkiye (114,000 tonnes). In percentage terms, these three countries accounted for 29 percent, 12 percent, and 6 percent of the world's total honey production, respectively, in that year. The largest EU producers were Spain, Romania, Germany, Hungary, and Poland, but, although EU honey production reached high levels of production, this was not enough to meet the high consumption of this foodstuff. In this matter, the EU honey market has been characterized by an imbalance between supply and demand, with more than half of the honey consumed being imported, as domestic honey production is only around 60 percent of the consumption rate. China, Ukraine, and several Latin American countries are the largest honey exporters to the EU, with Germany and the UK being the primary destinations, receiving around 60,000 and 45,000 tonnes per year, respectively ( Gabinete de Planeamento, Políticas e Administração Geral, 2020). According to data from the National Statistics Institute (INE), Portugal produced 11,465 tonnes of honey in 2022. On average, each resident consumed 1.3 kilograms out of a total of 14,000 tonnes consumed between 2022 and 2023 ( Instituto Nacional de Estatística, 2023).

## Honey: nutritional composition & functional properties

Honey is a complex food matrix composed of various nutrients and components. The percentage of each component can vary due to geographical and environmental conditions, the floral source used by the bees, the type of honeybee, and the extraction method used. Variations in honey, such as color, viscosity, taste, and properties, result from these conditions ( Ranneh *et al.*, 2021). For instance, its color ranges from almost colorless to dark brown and its consistency can be liquid, viscous or fully crystallized; its flavor and aroma are also determined by its plant source.

Honey is composed mainly of water and carbohydrates, with the latter accounting for approximately 80% of its total composition, together with organic acids, enzymes and solid particles obtained during honey collection. Monosaccharides, such as glucose and fructose, make up around 75% of the carbohydrate content ( Ranneh *et al.*, 2021). This nutrient is frequently linked to honey adulteration through the addition of fructose or glucose, which alters the sugar content and certain physical properties, such as the glass transition temperature ( Dranca *et al.*, 2022). Amino acids may also be found in honey, either in their free form or as part of proteins, accounting for approximately 0.5% of the total composition. Additionally, various minerals, such as calcium, chlorine, copper, iron, magnesium, phosphorus, potassium, sodium, and zinc, can be detected in honey in quantities ranging from 0.01 to 470 mg per 100 g ( Tafere, 2021). The honey's enzyme content can come from two types of bee metabolism: proteins and carbohydrates. Bees use various enzymes to digest proteins, such as trypsin, chymotrypsin, elastase, exopeptidase, and leucine aminopeptidases. For carbohydrate metabolism, examples of enzymes include diastase, invertase, glucosidase and glucose oxidase ( Babacan *et al.*, 2002; Chua *et al.*, 2015). Honey also contains phenolic compounds and flavonoids, which vary in quantity between different types of honey. These compounds are known for their antioxidant and antibacterial properties. Some of the specific compounds found in honey include gallic, ferulic, caffeic, chlorogenic, p-coumaric, syringic, and vanillic acids, as well as apigenin, catechin, luteolin, pinocembrin, galangin, and myricetin ( Young & Blundell, 2023).

When honey is sold or used in any product intended for human consumption, it must not contain any food additives or foreign organic or inorganic matter and must comply with the composition criteria ( European Commission, 2014) ( Table 1). Except for baker's honey, honey should not have any foreign tastes or odors, show signs of fermentation, have an artificially altered acidity, or have been heated in a way that destroys or significantly deactivates natural enzymes ( European Commission, 2014).

**Table 1.** Composition criteria of honey (adapted from European Commission, 2014).

Criteria	Type of honey and content
Fructose and glucose content (sum of both)	Blossom honey ≥ 60 g/100 g
	Honeydew honey, blends of honeydew honey with blossom honey ≥ 45 g/100 g
Sucrose content	In general, ≤ 5 g/100 g
	Specific varieties ≤ 10 - 15 g/100 g
Moisture content	In general, < 20 %
	Heather ( *Calluna*) and baker's honey in general: ≤ 23 %
	Baker's honey from heather ( *Calluna*) ≤ 25 %
Water-insoluble content	In general, ≤ 0,1 g/100 g
	Pressed honey ≤ 0,5 g/100 g
Free acid	In general, ≤ 50 milliequivalents per kg
	Baker’s honey ≤ 80 milliequivalents per kg
Diastase activity (Schade scale)	In general (except baker's honey) ≥ 8
	Honeys with low natural enzyme content and an HMF content of not more than 15 mg/kg ≥ 3
Hydroxymethylfurfural content (HMF)	In general (except baker's honey) ≤ 40 mg/kg
	Honeys of declared origin from regions with tropical climate and blends of these honeys ≤ 80 mg/kg

Honey has also been linked with various health benefits and functional properties such as antioxidant and antimicrobial activities. It is crucial to underscore that the functional properties and health benefits associated with honey can be attained through the consumption of pure, uncontaminated honey, without the application of rigorous technological processing or prolonged storage. This food has high potential as a natural antioxidant for foods due to its composition in phenolic compounds, namely phenolic acids and flavonoids, as well as other substances like catalase, carotenoids, organic acids, ascorbic acid, amino acids, and proteins. The mechanisms of antioxidant action reduce the adverse consequences of reactive oxygen and nitrogen species. This is achieved by inhibiting superoxide anion-producing enzymes, chelating metals, breaking down radical chain reactions, and preventing the formation of reactive oxidants ( Bogdanov *et al.*, 2008; Machado De-Melo *et al.*, 2018). On the other hand, its antimicrobial activity is directly linked with the high sugar content, low pH, the presence of hydrogen peroxide and polyphenolic compounds, as well as antimicrobial peptides. Recent studies have shown that this foodstuff exhibits effective antibacterial activity against aerobic, anaerobic, *Gram*-positive, and *Gram*-negative bacteria ( Olaitan *et al.*, 2007; Young & Blundell, 2023).

## Bee diseases

Honeybees are susceptible to infectious diseases introduced by bacteria, viruses, fungi, and parasites, which have contributed to the decline of colony populations and significant economic losses. Three diseases are often mentioned: American foulbrood (AFB), European foulbrood (EFB) and Nosemosis, whose causative agents are spore forming and non-spore-forming bacteria and fungus, respectively ( Applegate & Petritz, 2020).

The larval stage of the honeybee is vulnerable to AFB, a fatal disease caused by the *Gram*-positive, spore-forming bacterium *Paenibacillus* larvae. The endospores are the only infectious form of the organism and solely infect the larvae, as adult bees are not affected. Ingesting 10 or fewer spores from contaminated food is enough to cause death in larvae, with their susceptibility increasing as they age decreases. The germination of inactive spores into active vegetative cells occurs in response to stimuli such as L-tyrosine and uric acid, resulting in the rapid proliferation of P. larvae ( Daisley *et al.*, 2023; Genersch, 2010). Infection with the bacterium *Melissococcus plutonius* causes EFB, which mainly affects unsealed brood, resulting in the death of bee larvae when they are between 4 and 5 days old. Although older larvae are less affected by the infection, they are still susceptible to it. During the first phase, the larvae ingest food contaminated with bacterial cells, which colonizes their midgut asymptomatically. The infection enters a second phase that leads to tissue damage and a noticeable symptomatic phase. The pathogenic effect of EFB is caused by competition for nutrients between the infected larva and the pathogen. However, death may also result from other pathogenic mechanisms ( Forsgren, 2010). Both AFB and EFB diseases are similar in terms of symptoms but differ in their causative organisms. EFB is caused by the bacterium *M. plutonius*, which does not form spores and is therefore sometimes considered less problematic than AFB, whose spores from P. larvae can remain viable for years even under adverse conditions ( Reybroeck *et al.*, 2012). Nosemosis, also known as *Nosema* disease, has been identified as a major cause of declining adult honeybees’ health. This disease is caused by *Nosema ceranae*, an intracellular microsporidian intestinal parasite. Once ingested, the spores develop and multiply in the digestive tract, using energy and nutrients from the cells until they cause cell lysis. The spores are then released into the intestinal lumen, where they can infect adjacent cells. The spread of infection can be facilitated by the fact that spores can be shed from the host in feces, which in turn can contaminate neighboring honeycombs or food sources ( Ponkit *et al.*, 2021). Studies have shown that nosemosis is not always associated with colony weakness or bee mortality; this variability depends on factors such as parasite or host genetics, nutrition, climate or interactions with other environmental contaminants or parasites ( Paris *et al.*, 2018).

As AFB is a notifiable disease in many countries, its control and treatment are generally regulated by law and include the destruction of infected hives. The use of antibiotics to treat bee diseases is not permitted in most European countries. However, in countries where their use is permitted, the use of oxytetracycline or sulphathiazole is a common strategy for the prevention and treatment of infected colonies ( Genersch, 2010). Similarly, oxytetracycline is applied to colonies to prevent and treat EFB, but the emergence of *M. plutonius* resistant to this antibiotic has led to a reduction in its use ( Forsgren, 2010). Fumagilin is the only antibiotic treatment often mentioned for the control of Nosema disease, and recent studies have shown a temporary reduction in spore activity. However, the risk associated with the presence of residues of this antibiotic in products such as honey or royal jelly is of great concern ( Chaimanee *et al.*, 2021). In the context of bee health in Portugal, the law-decree n ^⍛^ 203/2005 defines the following diseases as compulsorily notifiable: American Foulbrood (AFB), European Foulbrood (EFB), Acarapisis, Varroasis, Aethinosis due to *Aethina tumida*, Tropilaelaps due to *Tropilaelaps* spp., Ascosferiosis (only in controlled areas) and Nosemiasis (only in controlled areas) ( Ministério da Agricultura, do Desenvolvimento Rural e das Pescas, 2005). The veterinary measures to be applied may include: (a) health inspections and investigations; (b) the demarcation of places or regions to be considered infected and the attribution of health status to geographical areas; (c) restrictions and conditions on the transit of bees, swarms, colonies or hives and products thereof, and substances or materials intended for use in apiculture, which may present a risk of introducing a compulsorily notifiable or exotic disease; (d) treatment, slaughter, hygiene and disinfection measures.

## Veterinary drugs: classification & chemistry

Antibiotics are among the most widely used drugs in both human and veterinary medicine. Their use may be for therapeutic or prophylactic purposes, or, in the case of food-producing animals, they may be administered with the aim of increasing growth and hence productivity. However, the excessive use of this type of medicine and its accumulation in the tissues of animals, and consequently in the food they produce, can have adverse effects on consumer health, including the emergence of antibiotic resistance in pathogenic microorganisms and the occurrence of allergic reactions in hypersensitive individuals. In this context, the European Commission has set a list of antibiotics and respective classes of antibiotics in alignment with OneHealth approach against antimicrobial resistance, including aminoglycosides, amphenicols, β–lactams, lincosamides, macrolides, nitrofurans, quinolones, sulfonamides, and tetracyclines.

### Aminoglycosides

**Figure 1.**
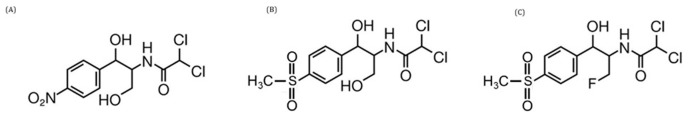
Structure of streptomycin ( **A**), neomycin ( **B**) and gentamicin ( **C**).

Aminoglycosides are one of the oldest classes of antimicrobials, with streptomycin being the first compound in this group to be introduced into therapy in 1944. Compounds in this class can be derived from *Streptomyces* spp. (e.g. streptomycin, neomycin and spectinomycin) and *Micromonospora* spp. (e.g. gentamicin) or from chemical synthesis. Structurally, there are two or more amino sugars linked by glycosidic bonds to the central nucleus - cyclitol. In Figure 1, examples of the chemical structure of this family of antibiotics are provided. Aminoglycosides are active against various *Gram*-positive and *Gram*-negative microorganisms, mycobacteria, and protozoa. They work by binding with high affinity to the A site of the 16S ribosomal RNA of the 30S subunit of the ribosome. This interaction can lead to mistranslation, which in turn can lead to errors in protein synthesis due to the incorrect binding of amino acids to a polypeptide in assembly. Some aminoglycosides have mechanisms of action that affect protein synthesis by blocking elongation or directly inhibiting initiation ( Durante-Mangoni *et al.*, 2009; Farouk *et al.*, 2015; Glinka *et al.*, 2020; Krause *et al.*, 2016; Perkons *et al.*, 2018).

Molecules in this class are highly hydrophilic, making it necessary to use ion-pairing reagents as part of the mobile phase to achieve sufficient retention on reversed-phase HPLC columns. Examples include sodium dodecyl sulphate, sodium heptanesulphonate, nonadluoropentanoic acid and HFBA. These types of reagents increase the hydrogen ion concentration in the eluent, which enhances positive ionization but suppresses negative ionization, making it difficult to develop multi-methods that include aminoglycosides and acidic compounds (e.g., chloramphenicol), which are efficiently ionized in the negative mode. Ion-pairing reagents can change the retention order of the compounds being analyzed, resulting in their possible co-elution ( Perin *et al.*, 2023; Tölgyesi *et al.*, 2018). Another reason for the difficulty of including aminoglycosides in multi-methods is that these analytes are lost when using solvents such as acetonitrile (ACN), or methanol (MeOH), as they remain in the aqueous fraction, where they have high solubility ( El Hawari *et al.*, 2017).

### Amphenicols

**Figure 2.**
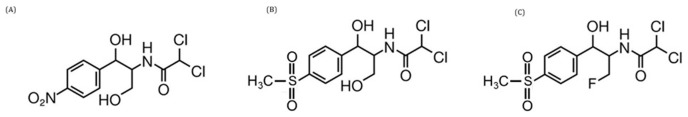
Structure of chloramphenicol ( **A**), thiamphenicol ( **B**) and florfenicol ( **C**).

Synthetic compounds belonging to the amphenicol family are active against *Gram*-positive and *Gram*-negative bacteria and can also be effective against anaerobic microorganisms and viruses that contain a phenylpropanoid structure. Their antibacterial action occurs after the antibiotics bind to bacterial ribosomal subunits, resulting in inhibition of the enzyme peptidyl transferase, which in turn reduces the transfer of amino acids to the peptide chain being formed and inhibits protein synthesis. This family includes small organic and fat-soluble molecules such as thiamphenicol, florfenicol and chloramphenicol ( Figure 2), which was first discovered and is widely used in human and veterinary medicine. However, its use in food-producing animals has been banned due to potential adverse effects on human health ( David *et al.*, 2022; Guidi *et al.*, 2017).

### β–Lactams

**Figure 3.**
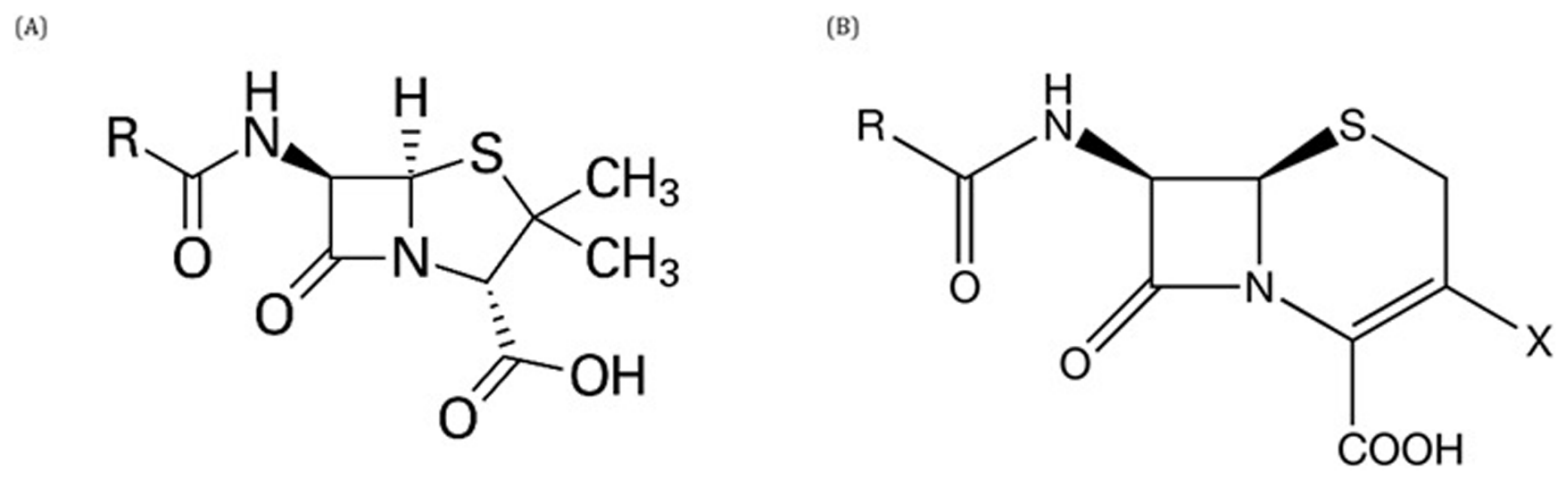
Basic structure of penicillins ( **A**) and cephalosporins ( **B**).

The β-lactam antibiotics are a class widely used in both human and veterinary medicine and can be divided into five subclasses based on variations in their structure: i) penicillins, ii) cephalosporins, iii) cephamycins, iv) monobactams and v) carbapenems. A β-lactam ring is present in the central part of their structure, and, except for the monobactams, an additional ring structure may be attached to it. Its bactericidal activity is based on the disruption of bacterial cell wall formation by covalently binding to enzymes involved in the final steps of peptidoglycan cross-linking in *Gram*-negative and *Gram*-positive bacteria. Inhibition of peptidoglycan synthesis or its degradation during bacterial growth results in a damaged bacterial wall, which is disastrous for the bacterial cell, as it may enter lysis. The two main groups with a bicyclic structure are the penicillins with 6-aminopenicillanic acid and the cephalosporins with 7-aminocephalosporanic acid ( Figure 3). The presence of the β-lactam ring in both groups makes them more susceptible to several degradation processes, for example in acidic media, and at room temperature, the penicillin ring undergoes reconfiguration ( Bush & Bradford, 2016; Fernandes *et al.*, 2013; Lima *et al.*, 2020; Sta Ana *et al.*, 2021).

### Lincosamides

**Figure 4.**
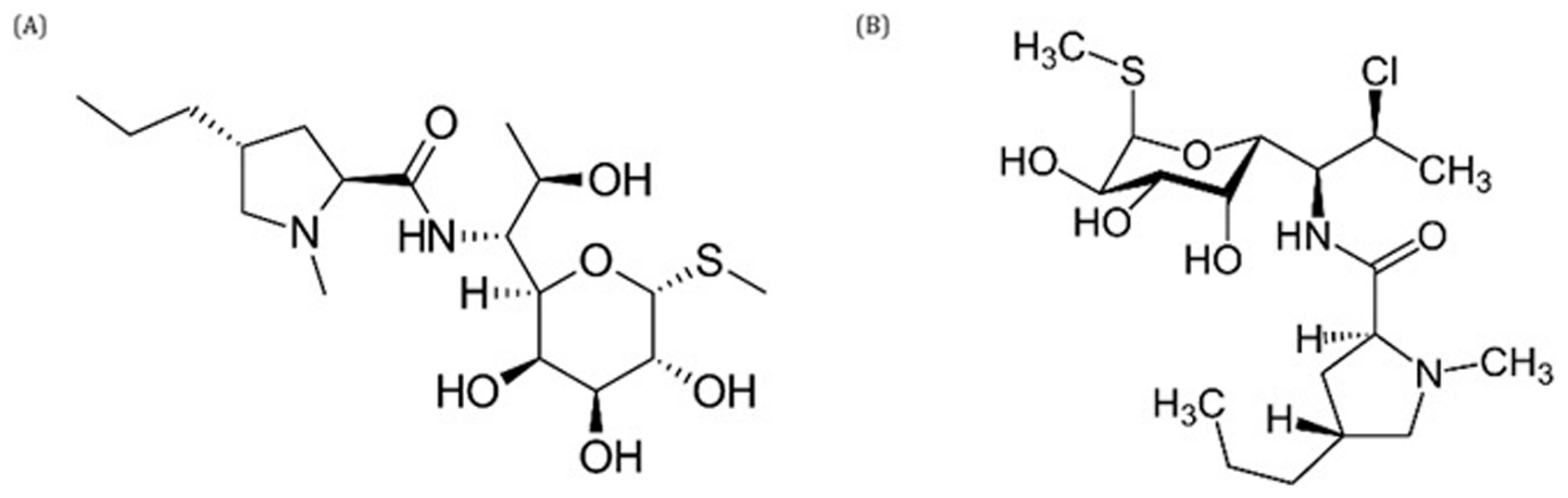
Structure of lincomycin ( **A**) and clindamycin ( **B**).

Lincosamides are a relatively small group of antibiotics with a chemical structure containing amino acids and sugar molecules. Naturally occurring lincomycin is a member of this class of compounds, and various semisynthetic derivatives have been synthesized from it, such as clindamycin ( Figure 4). This group of compounds is potentially and widely active against Gram-positive bacteria and some protozoa, interfering with protein synthesis. Their action against microorganisms is made possible by the structural similarity of these antibiotics to the 3' end of the L-Pro-Met of transfer RNA (tRNA), which allows them to bind to and interfere with the peptidyl transferase center present in the 50S subunit of the bacterial ribosome, causing disruption of protein synthesis at an early stage of amino acid chain elongation ( Spížek & Řezanka, 2017; Zhang *et al.*, 2018).

### Macrolides

**Figure 5.**
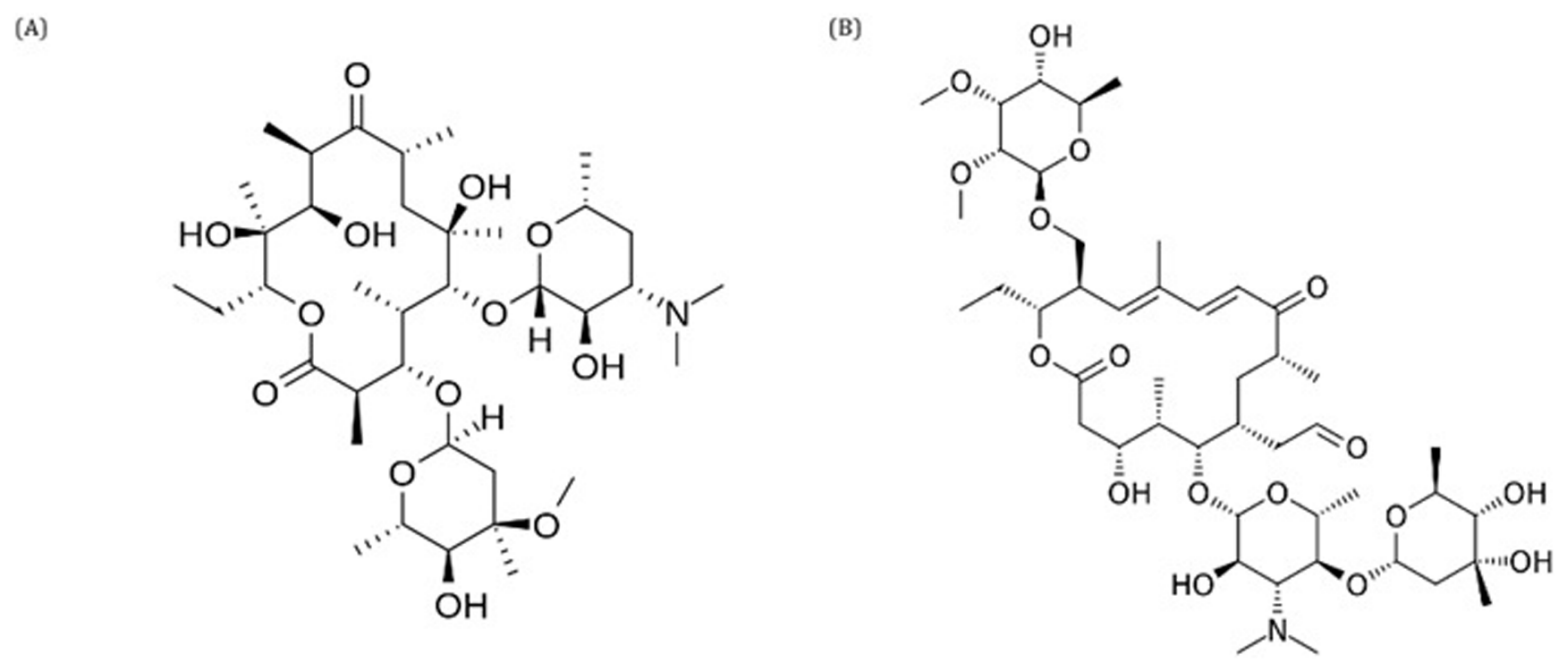
Structure of erythromycin ( **A**) and tylosin ( **B**).

Compounds in the macrolide class are widely used to treat infections caused mainly by gram-positive bacteria and, to a lesser extent by gram-positive bacteria and some anaerobic bacteria. Traditionally, it was thought that protein synthesis was inhibited after translation had stopped by blocking the exit channel of the newly formed polypeptides from the ribosomal subunit; however, studies have shown that antibiotics of the macrolide class are molecules that selectively modulate protein synthesis. The functional properties of the catalytic center of the ribosome are allosterically affected by antibiotics and newly formed peptides, and the binding of antibiotics to the ribosome prevents the polymerization of specific amino acid sequences. Structurally, they consist of a macrocyclic lactone ring which can contain between 12 and 16 members to which amino sugars and/or neutral sugar molecules can be linked by glycosidic bonds ( Figure 5). Most macrolides are synthesized by Streptomyces strains, but a significant number of Micromonospora species have been found to produce compounds of this class, with 14 or 16 members ( Dinos, 2017; Vázquez-Laslop & Mankin, 2018).

### Nitrofurans

**Figure 6.**
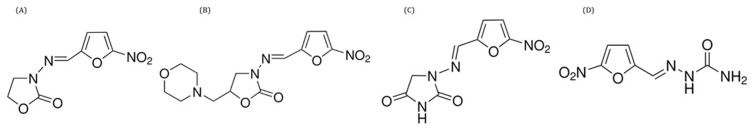
Structure of furazolidone ( **A**), furaltadone ( **B**), nitrofurantoin ( **C**) and nitrofurazone ( **D**).

Nitrofurans, in particular furazolidone (AOZ), furaltadone (AMOZ), nitrofurantoin (AHD) and nitrofurazone (SEM), are synthetic broad-spectrum antibiotics whose basic structure contains a 5-nitrofuran ring ( Figure 6). These compounds are rapidly metabolized after ingestion to form metabolites capable of binding to tissue proteins. This class of compounds has been widely used as feed additives for growth promotion and mainly in livestock, aquaculture, and bee colonies for prophylactic and therapeutic treatment of bacterial infections. In the EU, their use in farm animals was banned in 1995 because of the carcinogenicity and other adverse effects of residues of these antibiotics on human health.

The detection of nitrofuran metabolites poses some problems related to their metabolism and structure, such as the fact that they bind to food proteins, making it necessary to perform acid hydrolysis for their isolation, the fact that they are very hydrophilic, making it difficult to apply LLE and SPE, as well as their separation on reversed-phase columns, and the fact that they are low molecular weight compounds with no chromophores in their composition, which makes it necessary to perform a preliminary derivatization using, for example, 2-nitrobenzaldehyde (2-NBA), since their determination using UV detectors and mass spectrometry is not very sensitive ( Martos & Shurmer, 2012; Melekhin *et al.*, 2021; Vass *et al.*, 2008).

### Quinolones

**Figure 7.**
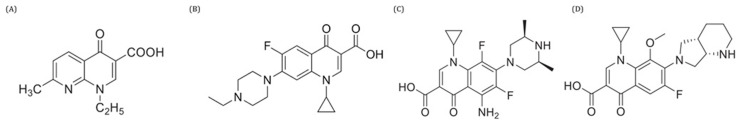
Structure of nalidixic acid ( **A**) enrofloxacin, ( **B**), sparfloxacin ( **C**) and moxifloxacin ( **D**).

Quinolones are chemically synthesized antibiotics with a broad spectrum of activity and, the 4 generations developed share a central bicyclic structure related to the compound 4-oxo-1,4-hydroxyquinolone (4-quinolone). The first generation (nalidixic and oxolinic acids) are active against *Gram*-negative bacteria. The fluoroquinolone subclass (2nd generation) emerged after the introduction of a fluorine atom and a piperidine group, resulting in a wider spectrum of activity against *Gram* -negative bacteria and some *Gram* -positive organisms. The third generation of this class of compounds was synthesized by substitutions in two positions of the skeletal structure, increasing activity against *Gram* -positive bacteria. The latest generation of quinolones has improved efficacy and a broader spectrum that includes anaerobic bacteria. DNA synthesis is inhibited by these compounds by disrupting bacterial type II topoisomerase or by inhibiting the catalytic activity of DNA gyrase and topoisomerase IV, both essential enzymes for regulating chromosomal supercoiling necessary for DNA synthesis. By binding to enzyme-DNA complexes, quinolones inhibit DNA replication, with consequences for cell division, culminating in cell death ( Cheng *et al.*, 2013; Dhiman *et al.*, 2019; Pan *et al.*, 2022; Pham *et al.*, 2019). Some examples of quinolones comprise nalidixic acid (1 ^st^ generation), enrofloxacin (2 ^nd^ generation), sparfloxacin (3 ^rd^ generation) and moxifloxacin (4 ^th^ generation) ( Figure 7).

### Sulfonamides and Trimethoprim

**Figure 8.**
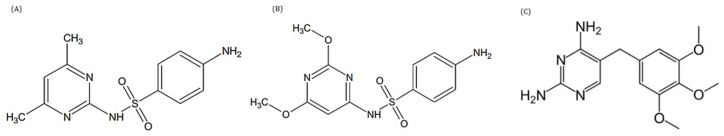
Structure of sulfamethazine ( **A**), sulfadimethoxine ( **B**) and trimethoprim ( **C**).

Compounds in the sulfonamides class are synthetic antimicrobial drugs used in the broad-spectrum pharmacological treatment of bacterial infections in humans and animals, such as, for example, sulfamethazine and sulfadimethoxine ( Figure 8. A and B). Structurally, they are organosulfur compounds containing a -SO _2_NH _2_ and/or -SO _2_NH group and a sulfanilamide group, as well as distinct 6- or 5-membered heterocyclic rings. Their antimicrobial activity is effective against *Gram*-positive and some *Gram*-negative bacteria. Sulfonamides are structural analogues of p-aminobenzoic acid (PABA), which is required for the synthesis of folic acid, which is essential to produce nucleic acids. The structural similarity allows the antibiotics to bind to dihydropteroate synthase, which inhibits the formation of dihydrofolate, tetrahydrofolate and subsequently DNA synthesis and bacterial cell division or replication. For synergistic and potentiating effects, some compounds in this class are used in combination with trimethoprim ( Figure 8.C), whose inhibitory action on the enzyme dihydrofolate reductase also interferes with folic acid metabolism, specifically the conversion of dihydrofolic acid to tetrahydrofolic acid.

The chemical structure of this class allows them to bind via N-glycosidic bonds to the reduced sugars present in honey, so the extraction process must include an acid hydrolysis step to break these bonds and release the sulfonamides. However, in acidic media, certain antibiotics, or classes, such as macrolides and tetracyclines, can suffer degradation, leading to poor recoveries, again making it difficult to use multi-class methods capable of extracting and detecting all the types of antibiotics being analyzed ( Dmitrienko *et al.*, 2014; Ovung & Bhattacharyya, 2021; Varenina *et al.*, 2023).

### Tetracyclines

**Figure 9.**
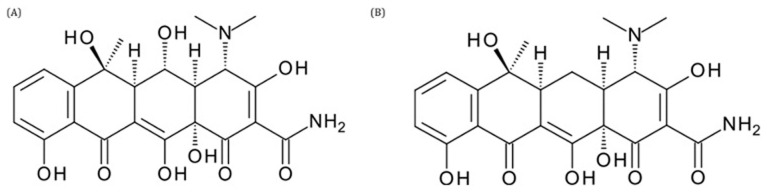
Structure of oxytetracycline ( **A**) and tetracycline ( **B**).

Tetracyclines have a very broad spectrum of activity, covering *Gram*-positive and *Gram*-negative bacteria, obligate intracellular bacteria and protozoan parasites. Chlortetracycline, produced by *Streptomyces aureofaciens*, was the first compound of this class to be isolated, followed by oxytetracycline and tetracycline ( Figure 9), among others. Compounds in this class can be synthesized naturally, produced by streptomyces or in the form of semisynthetic derivatives (metacycline, doxycycline, minocycline, etc.). The central structure is made up of four rings (A-D), one of which is aromatic, and the others are saturated, and because of the occurrence of various substitutions with different atoms or groups, 3 generations of this class of compounds have emerged. Their mechanism of action is based on the interaction of tetracyclines with bacterial ribosomes, specifically the 16S rRNA in the 30S subunit, interrupting translation by interfering with the aminoacyl-tRNA bond during the elongation necessary for protein synthesis.

Compounds in this class are relatively stable in acidic but not alkaline media. The polar nature of tetracyclines makes them prone to strong binding to proteins and chelation to divalent metal ions, which can negatively affect recovery during the extraction process. For this reason, tetracycline extraction protocols generally involve the use of acidic solvents with added metal chelating agents, such as Na _2_EDTA-McIlvaine buffer (pH = 4). Another issue related to their chemical nature is that they can undergo structural epimerization under acidic conditions (pH 2 to 6) ( El Hawari *et al.*, 2017; Grossman, 2016; Ramachanderan & Schaefer, 2021; Roberts, 2019).

## Honey and veterinary drugs

In beekeeping practice, antibiotics of different classes are used to control and treat the three main diseases affecting bees, which can be applied by feeding or spraying techniques. Among the most used are the aminoglycoside class (streptomycin and others), chloramphenicol, fumagillin, lincosamides (lincomycin), macrolides (erythromycin and tylosin), nitrofurans, nitroimidazoles, fluoroquinolones and quinolones, sulfonamides and tetracyclines ( Reybroeck *et al.*, 2012; Sharma *et al.*, 2023; Zhang *et al.*, 2019). Oxytetracycline has been the antibiotic of choice since the 1950s for the treatment of bacterial bee diseases. In general, in its hydrochloride form, it can be applied by four methods: paper packets of the antibiotic; spraying with powdered sugar containing oxytetracycline, repeated several times at weekly intervals; feeding the bees a solution of oxytetracycline in syrup; and, most commonly used nowadays, feeding an "extender patty" composed of oxytetracycline, sugar and vegetable fat ( Reybroeck *et al.*, 2012). The main problems associated with the extensive use of antibiotics are that these veterinary compounds are not effective against infectious spores, only suppressing clinical symptoms and masking AFB without curing it; chemical residues may persist in honey, affecting the quality and safety of this food for human consumption; and feeding antibiotics to larvae and adult bees can affect the vitality of the brood and the longevity of the bees. Finally, resistance to oxytetracycline and sulfathiazole in P. larvae has become widespread, necessitating the search for alternative treatments ( Genersch, 2010). Generally, the use of antibiotics is not permitted in this sector since residues have been found over the years in honey and royal jelly.

According to the Rapid Alert System for Food and Feed (RASFF), from 2020 to 2023, several notifications corresponding to the identification of residues of veterinary drugs in apiculture products (honey and royal jelly) were received, which included the presence of dapsone, nitrofuran, dihydrostreptomycin, tetracyclines, chloramphenicol, enrofloxacin, trimethoprim, ciprofloxacin ( European Commission, 2024) ( Table 2). Of the 388 notifications received between 2002 and 2022, about 79.64% concerned residues of veterinary medicines, in particular the sulfonamide class (sulfadimethoxine, sulfadimidine, sulfamethazine, sulfathiazole), nitrofurans (metabolite - nitrofurazone), aminoglycosides (streptomycin, dihydrostreptomycin), macrolides (tylosin, erythromycin), lincosamides (lincomycin), nitroimidazoles (metronidazole), quinolones (enrofloxacin, ciprofloxacin), tetracyclines (oxytetracycline) and chloramphenicol residues detected in honey samples from various countries including Argentina, Australia, Chile, China, Slovakia, Spain, Hungary, India, Israel, Italy, Lithuania, Poland, Portugal, Czech Republic, Romania, Turkey and Ukraine. The remaining notifications were related to adulteration/fraud (5.15%), the presence of foreign bodies (2.83%), pesticide residues (2.58%), and poor or insufficient controls (2.58%) ( Eissa & Taha, 2023).

**Table 2.** RASFF notifications from 2020 to 2023 (product category “honey and royal jelly”).

Date	Country	Origin of honey	Notification
9/01/2020	Czech Republic	Poland	Residue of prohibited substance Dapson in honey from Slovakia
20/05/2020	Belgium	Ukraine	Nitrofuran in honey
24/09/2021	Belgium	Belgium, China e Romania	Dihydrostreptomycin in acacia honey
18/02/2022	Belgium	Türkiye	Tetracyclines in honey from Türkiye
14/06/2023	Belgium	Romania	Chloramphenicol in honey from Poland (origin Romania)
19/07/2023	Germany	Mexico	Enrofloxacin and trimethoprim in honey from Mexico
14/08/2023	Slovakia	Czech Republic	Presence of ciprofloxacin in forest honey from the Czech Republic

### Regulatory frameworks

The legislation to be applied must be able to prevent residues of veterinary medicinal products (or residues of antibiotics) from entering the food chain and to control their levels to avoid negative effects on consumer health. The three pillars on which the application of legislation must be based are authorized products, maximum residue limits (MRLs), and guidelines for analytical methods ( Masiá *et al.*, 2016).

Regarding the first point, the EU legislative system distinguishes between medicines belonging to Group B, which includes authorized substances (aminoglycosides, lincomycin, macrolides, quinolones, sulfonamides and tetracyclines), and those belonging to Group A, which includes banned/prohibited substances (chloramphenicol, nitrofurans and nitroimidazoles) ( European Commission, 2003a). Commission Regulation (EU) n ^⍛^ 37/2010 sets the MRLs for antibiotics in food that must be verified for each substance before it can be used in food-producing animals such as bees. In the EU, there are no MRLs set for these antimicrobials in honey samples, which means that their use is not regulated for the prevention and treatment of diseases affecting honeybees ( European Commission, 2010; Simone *et al.*, 2017). Given that veterinary medicines are not classified as MRL substances in honey, a "zero tolerance" approach is required. In terms of analytical strategies, that situation leads to the need to have methods with limits of detection as low as possible. Concerning analytical methodologies, in the absence of harmonized rules or regulations, European Member States have defined different acceptable control methods, detection limits or sampling methods. Recommended Concentrations (RCs) for streptomycin, macrolides (erythromycin, tylosin), sulfonamides and tetracyclines in honey, listed in Table 3, have been proposed by the EU Reference Laboratories ( Community Reference Laboratories, 2007) and now updated for Minimum Method Performance Requirements (MMPRs) by the new guidelines ( European Union Reference Laboratories, 2022). The former RCs and the new MMPRs are used as reference in the development of methods because the detection capacity (CCβ) or decision limit (CCα) should be lower than the RCs and MMPRs. These antibiotics belong to group B of Annex I of Directive 96/23/EC and as such have fixed MRLs for foodstuffs other than honey ( Community Reference Laboratories, 2007; European Commission, 2010; European Union Reference Laboratories, 2022).

**Table 3.** Regulatory concentrations applied for antibiotics in honey in various countries (μg kg ^-1^).

	RC/MMPR	MRL						AL	NCL
Antibiotics	EU (RC)	China (MRL)	Japan (MRL)	USA	Canada (MRL)	Australia (MRL)	Switzerland (MRL)	Belgium (AL)	France (NCL)
Amoxicillin	-	-	8	-	-	-	-	-	-
Ampicillin	-	-	9	-	-	-	-	-	-
Chloramphenicol	0.15 (RPA)	ND	-	F	-	-	-	0.1 (MRPL)	-
Enrofloxacin	-	F	-	F	-	-	-	5	-
Erythromycin	20	ND	-	-	30 (RC)	-	-	20	-
Streptomycin	50	-	-	-	-	-	20	20	10
Fumagillin	-	-	-	-	25	-	-	25	-
Furazolidone	F	ND	-	F	-	-	-	-	-
Lincomycin	-	-	-	750	-	-	-	20	-
Nitrofurans	0.5 ^a^ (RPA)	-	-	-	-	-	-	1	-
Oxytetracycline	-	-	300	750	300	300	-	-	-
Sulfonamides	10	50	-	-	-	-	50	20	-
Tetracyclines	10	50	-	-	-	-	20	20	10
Tylosin	10	-	-	500	200	-	-	60	15

- Information not available RC - Recommended Concentration for searching missing info RPA – Reference Point for Action ND ND – Not detected AL – Action Limit NCL – Non-conformity Limit F – Forbidden substance ^a^ MRPL defined for poultry meat and aquaculture products applicable to honey. MMPR - Minimum Method Performance Requirements.

For banned substances (Group A) such as chloramphenicol and nitrofurans (furazolidone, furaltadone, nitrofurantoin and nitrofurazone), Minimum Required Performance Limit (MRPL) values are set at 0.3, and 1.0 μg/kg, respectively ( European Commission, 2003b; Zhang *et al.*, 2019). The setting of MRPLs was intended to harmonize the analytical performance of different methods and to ensure a similar level of consumer protection across the EU. The lack of regulatory harmonization has resulted in some European countries such as France setting non-compliance levels, Belgium setting action levels or MRPLs and recommended target concentrations, the United Kingdom setting non-compliance levels and Switzerland setting levels for certain antibiotics, as described in Table 3.

The Codex Alimentarius, established by the FAO and the World Health Organization, has also made it possible to establish harmonized international food standards to protect consumer health and promote fair practices in food trade. In the specific case of honey, this global standard does not set MRLs for antibiotics in this food. Similarly, in the USA, there are no tolerance levels, but the use of antibiotics such as oxytetracycline, tylosin and fumagillin is permitted. However, the use of these antibiotics in beekeeping has been reduced to avoid harmful residues in honey. In some countries, such as Canada, India and Argentina, the use of antibiotics such as oxytetracycline to treat AFB and EFB is still permitted, as is fumagillin to treat Nosema disease ( Reybroeck *et al.*, 2012; Simone *et al.*, 2017).

### Occurrence of antibiotic residues in honey

The extensive use of antibiotics in apiculture leads to an accumulation of antibiotic residues in honey. At a products’ perspective, a decreased on the quality of this food is observed, which endangers its marketing and on a consumers’ perspective, due to their long half-life, may have direct toxic effects ( Al-Waili *et al.*, 2012). Monitoring is therefore important in these matrices, and several international reports on the detection of antibiotic residues in honey samples have been published, in which antibiotic families of tetracyclines, sulfonamides, macrolides, lincosamides, fluoroquinolones, nitroimidazoles, and nitrofuran metabolites, have been reported, as shown in Table 4.

**Table 4.** Studies on the occurrence of antibiotics in honey samples.

Location	Number of samples	Positive results	Antibiotic Residue	Concentration level (μg/kg)	References
China	45	3	Metronidazole	5.87 – 66.95	( Lei *et al.*, 2018)
			Ciprofloxacin	52.91 – 89.43	
Georgia	49	3	Streptomycin	35.0 – 117.0	( Perkons *et al.*, 2018)
			Gentamycin C1	32.0	
Canada	35	12	Tylosin A	< LOQ – 0.0176	( von Eyken *et al.*, 2019)
			Tylosin B	0.0021 – 0.0703	
			Sulfamethazine	< LOQ	
			Sulfadimethoxine	< LOQ – 0.0074	
Türkiye	50	14	Quinolones	1.4 – 41.3	( İsmail Emir *et al.*, 2021)
China	28	1	Lincomycin	5.25	( Xu *et al.*, 2021)
China	85	5	Nitrofuran (metabolites)	1.20 – 3.36	( Yang *et al.*, 2022a)
Italy Hungary Romania Spain Serbia and Other countries	55	3	Sulfathiazole	0.5	( Paoletti *et al.*, 2022)
			Sulfamethazine	1.3	
			Tetracycline	0.5	
			Oxytetracycline	1.1	
China (Southeast)	50	7	Metacycline	159.9	( Yang *et al.*, 2022b)
			Oxytetracycline	4.0	
			Tetracycline	2.6 – 215.3	
			4-tetracycline	4.6 – 232.7	
Brazil	96	1	Enrofloxacin	< LOQ	( Perin *et al.*, 2023)

**LOQ** – Limit of Quantification.

Yang *et al.* (2022b) applied an extraction protocol to honey samples purchased from supermarkets, farmers' markets, convenience stores and online shops in different areas of southern China and found residues of metacycline (159.9 μg/kg), oxytetracycline (4.0 μg/kg), tetracycline (2.6 to 215.3 μg/kg) and 4-tetracycline metabolites (4.6 and 232.7 μg/kg) in the 50 samples analyzed. Another study in which antibiotics of the tetracycline’s family were detected, was performed by Paoletti *et al.* (2022), in a scope of 55 honeys purchased on local markets, mostly of European origin, including 29 from Italy, 7 from Hungary, 2 from Romania, 1 from Spain and 1 from Serbia, but also of unknown origin (4 honeys) or mixtures of honeys from different countries The search for 64 antibiotics yielded 3 positive results: one sample from Italy containing residues of oxytetracycline (1.1 μg/kg) and tetracycline (0.5 μg/kg), a mixed honey from Argentina, Moldova, Romania, Taiwan and Ukraine for the substance tetracycline (0.5 μg/kg), but also sulphamethazine (1.3 μg/kg), and a third one in a mixed honey from Hungary and Ukraine for the substance sulphathiazole (0.5 μg/kg). Sulfonamides were also identified in a Canadian study along with antibiotic residues of macrolides. von Eyken *et al.* (2019) obtained and analyzed 26 samples of honeys of different types (non-organic and organic, farmed, of different colours and floral origin) from different shops and farmers' markets in the Montreal and Calgary regions and 9 samples from the Canadian Food Inspection Agency (CFIA). Sulphamethoxine was the most frequently detected compound (eight positive samples), and in other two samples residues of sulphamethazine. Macrolide residues of tylosin A and B were detected in two samples, and only tylosin B in another sample. In this study, all positive results were at concentrations below the levels recommended in Canada. The presence of residues of antimicrobials of the macrolide and lincosamides groups, with a positive result for lincomycin (5.25 μg/kg) were also obtained by Xu *et al.* (2021) on a design study concerning 28 samples of honey commercialized from local markets.

Concerning fluoroquinolones in honey samples, several studies also comprised the positive detection of these veterinary drugs. For instance, Emir *et al.* (2021) selected honey samples from different origins (orange blossom, pine, chestnut, and citrus), and of the 50 samples analyzed, 14 tested positive and enrofloxacin was the most common antibiotic residue found (8 out of 50), followed by pipemidic acid (3 out of 50), danofloxacin (3 out of 50), ciprofloxacin (2 out of 50), lomefloxacin (1 out of 50) and cinoxacin (1 out of 50). In two separate samples, enrofloxacin (354.5 and 5.4 μg/kg) was detected together with ciprofloxacin (1.4 μg/kg) and pipemidic acid (3.2 μg/kg). Another honey sample contained ciprofloxacin (18.7 μg/kg) with danofloxacin (41.3 μg/kg), and another honey contained lomefloxacin (1.8 μg/kg) with danofloxacin. On another study comprising the analysis of 49 honey samples from different regions of Georgia, USA, residues of streptomycin were detected in two samples at concentration levels of 117 and 35 μg/kg, and of gentamicin C1 in another sample in a concentration of 32 μg/kg ( Perkons *et al.*, 2018). In a Brazilian study, 96 samples of honey were analyzed for the presence of 14 antimicrobials and only one residue of enrofloxacin at a concentration below the LOQ (< 5 μg/kg) was identified ( Perin *et al.*, 2023). Lei *et al.* (2018) analyzed 45 honey samples from honey producers and cooperatives in different cities in China and detected residues of metronidazole in three samples (66.95, 5.87 and 29.35 μg/kg) and ciprofloxacin in two samples (89.43 and 52.91 μg/kg). The nitrofurans’ class was also analysed by Yang *et al.* (2022a), which investigated the presence of these metabolites in 85 honeys obtained from the local market. Eighty samples were found to be negative, but antibiotic residues were detected in five honeys, including four positive results for aminurea (SEM) and one for 3-amino-2-oxalkyl ketone (AOZ) with concentrations of 3.36, 1.40, 1.20 and 2.64 and 2.35 μg/kg, respectively.

Other studies comprising the detection of antibiotic residues were found to be positive in real honey samples obtained from different forms of commerce, including convenience and online stores, supermarkets, local and agricultural markets, or directly from producers or producer cooperatives. The samples originated from countries such as China, Georgia, Canada, Türkiye, Brazil, and some European countries, namely Italy, Hungary, Romania, Spain, Serbia.

## Methods for extraction and detection of antibiotics in honey

The analysis of antibiotics in honey employs one of two principal methods, which are selected according to the objective of the determination. Multiclass analysis methods essentially employ confirmation methods, whereby an initial extraction step (section 6.1.) is conducted prior to the application of chromatographic methods coupled with mass spectrometry detection (section 6.2.). The second method type pertains to screening tests employing microbiological and immunoenzymatic techniques, which are capable of detecting the presence of an antibiotic or a family of antibiotics at a specific level. These include microbiological tests and enzyme-linked immunosorbent assays, which are typically referenced. The principal advantages of this approach are its speed, simplicity, and low cost. Nevertheless, a frequently cited disadvantage is that it can produce false positive results ( Guliy *et al.*, 2022; Jakšić *et al.*, 2018).

The development of extraction and detection methods for antibiotic residues in complex food matrices, such as honey, has become increasingly important, especially when it comes to multi-class methods that allow the presence and quantification of several residues belonging to more than one type of antibiotic family. The current trend is to develop methods that use fewer organic reagents, are less-time consuming, use low quantities of sample for analysis, and are more automated. However, the differences in the characteristics of the compounds belonging to the different families (e.g., chemical structure, polarity, solubility, stability conditions) and the complex matrix of honey make it difficult to establish an extraction and subsequent detection method capable of analyzing all of them with high precision and accuracy. In the next sections, extraction procedures, and analytical techniques for detection of antibiotic residues in honey will be discussed.

### Extraction methods

The choice of strategy for sample treatment should be based on the characteristics of the sample matrix, the physicochemical properties of the analytes, and procedures that may already have been developed and described in the literature. The hydrophilic nature of honey means that extraction starts with dissolution in ultrapure water, acidified solutions or even buffers ( Zhang *et al.*, 2019). For honey matrices, the most commonly used methods for the extraction of antibiotic residues can be divided into three types: solid-phase extraction (SPE), dispersive solid-phase extraction (d-SPE), liquid-liquid extraction (LLE), QuEChERS method and, finally, simpler methods consisting of dilution and injection of the sample. Some examples of each extraction methods are reported in Table 5 to Table 7.

**Table 5.** Multi-class confirmation methods for antibiotic residue analysis in honey - SPE, dSPE and LLE.

Compounds *	Sample weight (g)	Extraction / Clean-up	LC conditions		Detection	CCβ or LOD (μg/kg)	References
			LC Column	Mobile phase			
**Amphenicols (4)** **Cephalosporins (3)** **Lincosamides (1)** **Macrolides (3)** **Neonicotinoids (6)** **Nitrofurans (3)** **Nitroimidazoles (3)** **Penicillins (3)** **Quinolones (7)** **Sulfonamides (6)** **Tetracyclines (4)** **Others (1)**	1	TCA 20% McIlvaine buffer (pH 4.0) Oasis HLB - SPE	Hydro-RP (150 × 2.0 mm, i.d. 4 μm), with C18 guard column (4 × 3.0 mm;	0.1% FA in H _2_O / MeOH	LC – HRMS (Orbitrap) (ESI ±)	0.73 – 3.58	( Chiesa *et al.*, 2018)
**Aminoglycosides** **(14)**	1	*Extraction buffer:* Ammonium acetate 10 mM / Na _2_EDTA 0.4 mM/ 1% NaCl/ TCA 2% NaOH ( *pH * *adjustment*) HLB - SPE	Synergi Hydro-RP (150 × 2 mm, i.d. 4 μm) with C18 guard column (4 × 3 mm)	0.1% FA in H _2_O / MeOH	LC – HRMS (Orbitrap) (ESI ±)	0.12 – 5.56	( Bonerba *et al.*, 2021)
**Amphenicols (4)** **β - Lactams (20)** **Lincosamides (1)** **Macrolides (9)** **Nitroimidazoles (4)** **Pleuromutilins (2)** **Quinolones (22)** **Sulfonamides (9)** **Tetracyclines (4)**		TCA 20% McIlvaine buffer (pH 4.0) n - Hexane Oasis HLB - SPE					
**Aminoglycosides (7)** **Lincosamides (1)** **Quinolones (12)** **Sulfonamides (9)** **Tetracyclines (8)** **Others (3)**	5	0.1% (v/v) HFBA in H _2_O Strata - XL	Kinetex XB C-18 (100 mm × 3 mm, 2.6 μm)	0.1% HFBA in H _2_O / ACN	HPLC – MS/ MS (ESI +)	1 – 75	( Tölgyesi *et al.*, 2018)
**Nitroimidazoles (9)** **Quinolones (7)** **Sulfonamides (14)**	2	HCl 2M H _2_O n - Hexane Strata X-C (200 mg, 6 mL)	Poroshell 120 EC-C18 (100 × 3.0 mm; 2.7 μm) equipped withguard column (2.1 × 5 mm)	(a) H _2_O / ACN (b) 0.1% FA em H _2_O / MeOH ^a^	HPLC – MS/ MS (Q-TOF) (ESI ±)	0.1 – 9.8	( Paoletti *et al.*, 2022)
**Amphenicols (3)** **Lincosamides (2)** **Macrolides (13)** **Pleuromutilins (2)** **Quinolones (3)** **Tetracyclines (7)** **Others (3)**		McIlvaine buffer (pH 4.0) with 0.1M Na _2_EDTA Strata X (200 mg, 6 mL)					
**Aminoglycosides (6)**	5	TCA 1% NaOH 1M (pH adjustment) Strata-X PRP (200 mg/ 3 mL)	Obelisc R (2.1 × 150 mm, 5 μm)	0.1% FA in H _2_O / ACN / H _2_O	HPLC – MS/ MS (Q-TOF) (ESI +)	13.4 – 47.9	( Perkons *et al.*, 2018)
**Aminoglycosides (3)** **Lincosamides (1)** **Macrolides (6)** **Sulfonamides (6)** **Tetracyclines (8)**	2	H _2_O acidified MeOH (2M HCl) Na _4_EDTA (pH adjustment) PSA (d-SPE)	Zorbax SB C18 RP (100 × 2.1 mm; 3.5 μm)	HFBA 100 mM / ACN / H _2_O	HPLC – MS/ MS (ESI +)	7 – 13	( El Hawari *et al.*, 2017)
**Aminoglycosides (2)** **Amphenicols (1)** **Fluoroquinolones (2)** **Macrolides (2)** **Sulfonamides (3)** **Tetracyclines (4)**	2	H _2_O MeOH Na _2_-EDTA PSA (d-SPE)	Venusil XBP (100 mm × 2.1 mm, 3 μm) coupled to C18 guard column	(a) 0.1% FA in H _2_O / 0.1% ACN (b) HFBA 5M + Ammonium Acetate 5M / ACN w/ 5M + Ammonium Acetate 5M; (c) Ammonium Acetate 2M / ACN w/ Ammonium Acetate 2M ^b^	LC–MS/ MS (ESI ±)	0.15 – 5	( Perin *et al.*, 2023)
**Amphenicols (2)** **Nitrofurans (4)**	3	1) HCl 0.125M; 2-NBA 100mM (2) K _2_HPO _4_1M (pH adjustment) (3) NaCl; Ethyl acetate ^d^	XDB-C18 RP (50 × 4.6 mm, 1.8 μm)	(a) 0.1% FA in 8.5 mM Ammonium Acetate / MeOH (b) H _2_O / ACN ^e^	HPLC-MS/ MS (Qtrap) (APCI +) (ESI -)	0.189 – 2.35	( Veach *et al.*, 2018)

*In brackets: (number of compounds being analyzed) ACN – Acetonitrile; APCI – Atmospheric-Pressure Chemical Ionization; d-SPE – dispersive-Solid Phase Extraction; EDTA – Ethylenediaminetetraacetic acid; ESI ± - Electrospray in positive mode (+) / Electrospray in negative mode (-); FA – Formic Acid; HFBA – Heptafluorobutyric Acid; HLB – Hydrophilic-Lipophilic Balanced; HPLC-MS/MS – High-Performance Liquid Chromatography–tandem Mass Spectrometry; LC-HRMS – Liquid Chromatography–High Resolution Mass Spectrometry; LC-MS/MS – Liquid Chromatography tandem Mass Spectrometry; MeOH – Methanol; PSA – Primary and Secondary Amine; Q-TOF – Quadrupole Time-of-Flight; SPE – Solid Phase Extraction; TCA – Trichloroacetic Acid. ^a^ (a) Chloramphenicol; (b) Remaining antibiotics ^b^ (a) Tetracyclines, Fluoroquinolones, Macrolides, Sulfonamides; (b) Aminoglycosides; (c) Amphenicols ^c^ Adapted from ( El Hawari *et al.*, 2017) ^d^ Modified Liquid-Liquid Extraction (LLE) protocol ^e^ (a) Nitrofuran (metabolites); (b) Chloramphenicol and Florfenicol

**Table 6.** Multi-class confirmation methods for antibiotic residue analysis in honey - QuEChERS.

Compounds*	Sample weight (g)	Extraction / Clean-up	LC conditions		Detection	CCβ or LOD (μg/kg)	References
			LC Column	Mobile phase			
**Fluoroquinolones (9)** **Lincosamides (1)** **Macrolides (3)** **Sulfonamides (20)** **Tetracyclines (3)** **Others (1)**	2	Acetic acid 1% in ACN:H _2_O (80:20, v/v) Na _2_SO _4_ NaCl Bond Elut dSPE	Acquity UHPLC HSS T3 (1.8 μm, 2.1 × 150 mm)	0.1% FA in H _2_O / MeOH	UHPLC – MS/MS (ESI +)	0.1 – 2.5	( Varenina *et al.*, 2023)
**Lincosamides (2)** **Macrolides (13)**	2	Phosphate buffer (pH 8.0) ACN Na _2_SO _4_ ZnO	Waters XBridge C18 (150 mm × 2.1 mm i.d., 5 μm p. s.)	0.1% FA in H _2_O with 5 mM Ammonium Acetate / ACN	HPLC – MS/ MS (ESI +)	0.80 – 2.27	( Xu *et al.*, 2021)
**β - Lactams (14)** **Macrolides (9)** **Nitroimidazoles (1)** **Quinolones (15)** **Sulfonamides (22)** **Tetracyclines (9)**	2	Formic acid 0.1% in ACN : H _2_O (80:20, v/v) Na _2_EDTA Discorevy DSC-18	ACQUITY UPLC BEHC18 (100 mm × 2.1 mm, 1.7 μm)	MeOH / 0.2% FA in H _2_O	UHPLC – MS/MS (ESI +)	0.17 – 3.40	( Yang *et al.*, 2022b)
**Nitroimidazoles (3)** **Quinolones (3)**	3	McIlvaine buffer (pH 4.0) Citric acid : ACN (5:95) NaCl Na _2_SO _4_ PSA + C18 + Mg _2_SO _4_	Waters XTerra RP18 (2.1mm× 150 mm, 5 μm)	ACN / Ammonium formate 10 mM + 0.5% FA em H _2_O	LC -MS/MS (ESI (±)	2.13 – 5.27	( Lei *et al.*, 2018)
**Nitrofuran (4)**	4	2-NBA 0.1M HCl 2M K _3_PO _4_0.3M (pH adjustment) ACN NaCl PSA + C18	C18 (150 mm × 2.1 mm id, 5 μm particle size)	0.1% FA in H _2_O with 5 mM Ammonium Acetate / ACN	HPLC – MS/ MS (ESI +)	0.5	( Yang *et al.*, 2022a)
**Quinolones (21)**	5	H _2_O Ácido Acético 1% em ACN NaOAc; MgSO _4_ (DisQuE QuEChERS kit) MgSO _4_ + PSA	Waters ACQUITY BEH C18 (2.1 mm x 50 mm, 1.7 μm).	H _2_O; 0.5mM ammonium formate; 0.005% FA / MeOH 0.005% FA	UHPLC-MS/ MS (ESI +)	0.6 – 0.75	( İsmail Emir *et al.*, 2021)

ACN – Acetonitrile; d-SPE – dispersive-Solid Phase Extraction; ESI ± - Electrospray in positive mode (+) / Electrospray in negative mode (-); FA – Formic Acid; HPLC-MS/MS – High-Performance Liquid Chromatography–tandem Mass Spectrometry; LC-MS/MS – Liquid Chromatography tandem Mass Spectrometry; MeOH – Methanol; PSA – Primary and Secondary Amine; QuEChERS – "Quick, Easy, Cheap, Effective, Rugged, and Safe" method; UHPLC-MS/MS – Ultra-High Performance Liquid Chromatography tandem Mass Spectrometry.

**Table 7.** Multi-class confirmation methods for antibiotic residue analysis in honey - SLE.

Compounds*	Sample weight (g)	Extraction / Clean-up	LC conditions		Detection	CCβ or LOD (μg/kg)	References
			LC Column	Mobile phase			
**Lincosamide (1)** **Macrolides (2)** **Nitrofuran (1)** **Sulfonamides (3)**	0.2	ACN : H _2_O (50:50, v/v) H _2_O	InfinityLab Poroshell 120 Phenyl Hexyl (3.0 × 100 mm, 2.7 mm) with InfinityLab Poroshell 120 EC-C18 (3.0 × 5 mm, 2.7 mm) guard column	0.1% FA in H _2_O / MeOH	HPLC-Q-TOF-MS (DAD) (ESI +)	1.1 – 8.4	( von Eyken *et al.*, 2019)
		HCl 2M H _2_O					
**Quinolones (21)**	1	McIlvaine buffer - EDTA (pH 6.0)	Waters ACQUITY BEH C18 (2.1 mm × 50 mm, 1.7 μm).	H _2_O; 0.5mM ammonium formate; 0.005% FA / MeOH; 0.005% FA.	UHPLC-MS/MS (ESI +)	1.21 – 1.39	( İsmail Emir *et al.*, 2021)

ACN – Acetonitrile; DAD – Diode Array Detector; ESI ± - Electrospray in positive mode (+) / Electrospray in negative mode (-); FA – Formic Acid; HPLC-Q-TOF-MS – High Pressure Liquid Chromatography coupled with Quadrupole Time-of-Flight Mass Spectrometry; MeOH – Methanol; UHPLC-MS/MS – Ultra-High Performance Liquid Chromatography tandem Mass Spectrometry.

SPE is currently one of the most widely used purification techniques for trace analysis, as an alternative to LLE. The selectivity of this technique depends on the strength of the interaction between the analytes and the functional groups on the surface of the adsorbent, and these interactions can be hydrophobic, hydrophilic, or cationic-anionic. The most common adsorbents are classified as non-polar or reversed phase, polar or normal phase, ion exchange and immunoaffinity ( Badawy *et al.*, 2022). Reversed phase adsorbents are the most widely used for honey purification for most classes of antibiotics and are divided into two types of materials based on alkyl silica, with functional groups such as octyl (C8), octadecyl (C18) and phenyl (Ph) attached to the silica surface; and polymers such as hydrophilic-lipophilic balance (HLB), Strata-X (surface modified styrene-divinylbenzene), LiChrolut EN (highly cross-linked polystyrene-divinylbenzene) and Evolute ABN (cross-linked polystyrene-divinylbenzene functionalized with oligomeric hydroxyl groups), among others. The analytes are retained by the forces of attraction between their C-H bonds and the functional groups on the silica surface and are eluted using a solvent that is non-polar with respect to water, which breaks the bonds formed ( Pyrzynska, 2011). The fact that honey is a water-soluble matrix provides the use of non-polar adsorbents used in reversed phase conditions, as this is the technique of choice for the extraction of polar or hydrophobic organic analytes from aqueous or non-polar matrices. The d-SPE technique is a variation of SPE in which there is no retention of the analytes of interest as they remain in the matrix while the co-extractive compounds are retained on the adsorbent. LLE is used as the initial sample preparation step in various protocols and is based on the transfer of analytes from the sample (aqueous matrix) to a water-immiscible solvent such as ethyl acetate, dichloromethane, and chloroform ( Simone *et al.*, 2017). Most recently, the QuEChERS (quick, easy, cheap, effective, rugged, and safe) method has become more widely used for the analysis of pesticides and other types of contaminants in food and can be described as a variation of LLE followed by a d-SPE step. The method consists of two steps: in the first, the homogenized sample is extracted and partitioned using a solution of ACN and salts (MgSO _4_ and NaCl), and then part of the supernatant is purified using the d-SPE technique; the second step consists of purification using d-SPE and is sometimes omitted ( Musarurwa *et al.*, 2019). 

***Sample preparation*** In the case of complex food matrices such as honey, sample preparation is of paramount importance, as extensive purification of these matrices is necessary for the achievement of low detection limits and adequate selectivity. Different processes are used in this step, namely in what concerns initial sample weight (g), and sample pre-treatment, which can include dilution and homogenization, hydrolysis, or derivatization. The sample weight used for honey studies varies from 0.2 g to 5 g, with most studies reporting the use of 2 g for the practical work. As described in Table 5, when the extraction method was based on SPE, d-SPE or LLE techniques, an initial treatment was applied to the samples that included dilution and/or acidification of the honey with solvents such as 20% TCA and McIlvaine buffer (pH 4.0) ( Bonerba *et al.*, 2021; Chiesa *et al.*, 2018), 2M HCl in H _2_O ( Paoletti *et al.*, 2022), McIlvaine buffer (pH 4.0) with 0.1 M Na _2_EDTA ( Paoletti *et al.*, 2022), 0.1% HFBA in H _2_O ( Tölgyesi *et al.*, 2018), H _2_O and acidified MeOH (2M HCl) ( El Hawari *et al.*, 2017), H _2_O and MeOH ( Perin *et al.*, 2023). In some analytical studies, n-hexane was added during the extraction process for defatting purposes ( Bonerba *et al.*, 2021; Paoletti *et al.*, 2022).

As additional steps, some of the protocols include a pH adjustment to values of 2, 5.0 ± 1.0 and 7.3 ± 0.3 using solutions of Na _4_-EDTA ( El Hawari *et al.*, 2017), NaOH ( Perkons *et al.*, 2018) and K _2_HPO _4_ ( Veach *et al.*, 2018), respectively. A protein precipitation is often used by adding 20% and 1% TCA solution to honey samples ( Chiesa *et al.*, 2018; Perkons *et al.*, 2018).

Most recently, the QuEChERS method has been chosen for the extraction of antibiotic residues from honey. Briefly, a pre-treatment is applied with an initial dilution of the sample with a solution of phosphate buffer (pH 8.0) ( Xu *et al.*, 2021), H _2_O ( Emir *et al.*, 2021) or McIlvaine buffer (pH 4.0) ( Lei *et al.*, 2018). Regarding the extraction solvent, the authors chose solutions of 1% acetic acid in ACN:H _2_O (80:20, v/v) ( Varenina *et al.*, 2023), ACN ( Xu *et al.*, 2021; Yang *et al.*, 2022a), 0.1% formic acid in ACN:H _2_O (80:20, v/v) ( Yang *et al.*, 2022b), 1% acetic acid in ACN ( Emir *et al.*, 2021) and citric acid:ACN (5:95, v/v) ( Lei *et al.*, 2018). In the studies concerning the analysis of nitrofuran metabolites, a hydrolytic derivatization of the sample is commonly used by the addition of 2-NBA and HCl to the same solution ( Veach *et al.*, 2018; Yang *et al.*, 2022a). In the work by von Eyken *et al.* (2019), a simpler extraction procedure was used based on diluting the honey sample with ACN:H _2_O solution (1:1, v/v) (protocol A) or with an HCl solution (protocol B), followed by filtration through 0.22 μm PTFE, ending with further dilution in water until the final concentration equivalent to 1% honey. Similarly, Emir *et al.* (2021) added McIlvaine - EDTA buffer solution (pH 6.0) to the sample and filtered it through a 0.45 μm PVDF filter.

### Cleaning/purification methods

Extraction procedures are necessary to isolate and concentrate veterinary drug residues because they are found in complex food matrices at very low concentrations, as in the case of honey. Nowadays, the more traditional methods have been replaced by more "environmentally friendly" ones, which tend to simplify sample preparation, reduce the volume of solvents and the quantity of reagents considered toxic, and shorten the analysis time ( Masiá *et al.*, 2016).

In the clean-up phase, SPE cartridges allow the separation of the compounds of interest from the matrix, and different sorbents have been reported for honey analysis, including Oasis HLB ( Bonerba *et al.*, 2021; Chiesa *et al.*, 2018), Strata X-C (200 mg, 6 mL) ( Paoletti *et al.*, 2022), Strata-X polymeric RP (200 mg, 3 mL) ( Perkons *et al.*, 2018), and Strata Xl ( Tölgyesi *et al.*, 2018), and, in the case of the d-SPE method, PSA reagent ( El Hawari *et al.*, 2017; Perin *et al.*, 2023) ( Table 5). For QuEChERS protocols, extraction salts mostly used are Na _2_SO _4_ ( Lei *et al.*, 2018; Varenina *et al.*, 2023; Xu *et al.*, 2021) and NaCl ( Lei *et al.*, 2018; Varenina *et al.*, 2023; Yang *et al.*, 2022a), Na _2_EDTA ( Yang *et al.*, 2022b) and NaOAc and MgSO _4_ ( İsmail Emir *et al.*, 2021). In the clean-up step, sorbents such as Bond Elut d-SPE salts ( Varenina *et al.*, 2023), ZnO ( Xu *et al.*, 2021), PSA + C18 ( Yang *et al.*, 2022b), Discovery DSC-18 ( Yang *et al.*, 2022b), MgSO _4_ + PSA ( Emir *et al.*, 2021), PSA + C18 + anhydrous MgSO _4_ ( Lei *et al.*, 2018) were added ( Table 6).

### Separation and detection methods (analytical techniques)

Residues in food are currently analyzed using liquid chromatography-mass spectrometry (LC-MS). Analysis of more complex samples has become possible with the introduction of triple quadrupole mass spectrometers (LC-MS/MS or LC-QqQ) using the multiple reaction monitoring (MRM) detection method, which has increased the selectivity and precision of the methods, improved the signal-to-noise ratio (S/N), lowered the limits of quantification, and increased the range of linearity ( Zhang *et al.*, 2019). LC systems coupled with high-resolution mass spectrometry (LC-HMRS) have also emerged as an alternative to LC-MS/MS and have made it possible to increase the selectivity and sensitivity of the equipment. The two HMRS technologies include time-of-flight (ToF), and Orbitrap and are commonly used in protocols for the analysis of residues in honey ( Krauss *et al.*, 2010). The ionization source is generally electrospray in positive mode (ESI+) for most antibiotic groups and in negative mode (ESI-) for chloramphenicol ( Paoletti *et al.*, 2022). As far as chromatographic separation is concerned, liquid chromatography is used instead of gas chromatography because antibiotics are polar in nature, have low volatility and are more unstable, making them unsuitable compounds for analysis by GC ( Bobbitt & Ng, 1992). Technological advances introduced ultra high-performance liquid chromatography (UHPLC), which is currently the separation technique used to analyze antibiotics in food matrices. UHPLC has emerged from continuous improvements in chromatographic techniques and can also be used for trace studies in various matrices ( Aguilera-Luiz *et al.*, 2013). In general, chromatographic separation is performed using reversed-phase columns, except for the aminoglycoside family (e.g., streptomycin and dihydrostreptomycin) where hydrophilic interactions chromatography (HILIC) columns are frequently applied ( Perkons *et al.*, 2018; Simone *et al.*, 2017). These separation and detection methods are the most commonly reported in literature for the analysis of antibiotic residues in honey, as shown in Table 5 to Table 7.

### Method validation

The Commission Implementing Regulation (EU) 2021/808 of 22 March 2021 defines the requirements for validation of analytical method for residues of pharmacologically active substances used in food-producing animals and on the interpretation of results as well as on the methods to be used for sampling. In this regulatory framework, performance criteria are defined as according to two categories of analytical methodologies: screening or confirmation, which are further subdivided into qualitative, semi-quantitative and quantitative, and qualitative and quantitative ( European Commission, 2021). Screening methods are used to screen a substance or class at the required level. A confirmation method, on the other hand, provides complete or complementary information for the unequivocal identification of pharmacologically active substances and, if necessary, its quantification in one of the following ways: (a) at the MRL for authorized substances; (b) at the reference value for action in the case of prohibited or unauthorized substances for which a reference value for action has been established; and (c) at a concentration as low as reasonably achievable in the case of a prohibited or unauthorized substance for which no reference value for action has been established. For the correct interpretation of results, it is necessary to take into account the definition of concepts related to confirmation methods, such as the Confirmation Decision Limit, or CCα, which is defined in the Regulation as "the limit at which and from which it can be concluded, with a probability of error α, that a sample is non-compliant, where the value 1 - α represents the statistical certainty, expressed as a percentage, that the permitted limit has been exceeded", where a result is considered non-compliant if it is equal to or greater than the CCα. The alpha error (α) defines the "probability that the analyzed sample is compliant even though a non-compliant measurement result has been obtained" and should be 1% or less for unauthorized or prohibited substances and 5% or less for others. In the case of screening methods, the CCβ or detection capacity for screening, is defined as "the lowest analyte content that can be detected or quantified in a sample with a probability of error of β" and the beta error (β) as "the probability that the analyzed sample is in fact non-compliant, although a compliant measurement result has been obtained", which must be equal to or less than 5%. For methods used to analyze prohibited or unauthorized substances, the CCα value (confirmation method) and the CCβ value (screening method) should be as low as reasonably achievable. In the case of the analysis of authorized substances, the CCα value obtained should be higher than, but as close as possible to, the MRL or ML and, in the case of CCβ, lower than the MRL or ML. Confirmation methods shall comply with the criteria applicable to the relevant performance characteristics, which are CCα, trueness, precision, relative matrix effect/absolute recovery, selectivity/specificity, stability, and robustness (quantitative) or only CCα and general requirements (qualitative). The requirements to be checked in the case of a screening method are CCβ, accuracy (quantitative), precision (semi-quantitative and quantitative), selectivity/specificity, stability, and robustness.

## Conclusions

Honey is one of the many products derived from beekeeping, and its production is ensured by extensive colonies spread throughout the world, so it is essential that the health of the colonies is guaranteed. Beekeeping is of great importance to other sectors such as agriculture for pollination and food production, producing tons of honey every year. Bee farms have suffered declines in colony populations, resulting in significant economic losses, often due to bacterial diseases such as American Foulbrood, European Foulbrood and Nosema. Continued and extensive use of antimicrobials in bees, either prophylactically or therapeutically, could lead to the development of resistance in their microorganisms to pathogens and the accumulation of drug residues in the honey they produce. Excessive consumption of these compounds by consumers could lead to adverse effects, such as allergic reactions.

About the authorization of the use of medicinal products in animals, EU legislation distinguishes between authorized (Group B) and prohibited (Group B) substances. MRLs for antibiotics in food are laid down in Commission Regulation (EU) n ^⍛^ 37/2010, but they are not defined for honey. The lack of legislative harmonization has led some countries to establish recommended concentrations and minimum performance limits for residue testing methods. The RASFF system allows for the rapid exchange of information and implementation of measures when the presence of contaminants in food or feed is reported. The antibiotics most frequently found in honey products in recent years are aminoglycosides, chloramphenicol, lincosamides, macrolides, nitroimidazole, quinolones, sulfonamides, tetracyclines and nitrofuran metabolites. Following a review of the literature, the search for antibiotic residues in real samples of honey from different origins gave positive results for compounds belonging to the same families as those reported in the RASFF system.

The most frequently used extraction methods include solid-phase extraction, dispersed solid or liquid (SPE, d-SPE or LLE), the QuEChERS method or simpler sample dilution and injection methods. The complexity of the honey matrix requires an initial treatment such as dilution or acidification of samples with McIlvaine buffer, H _2_O, MeOH or acidified ACN and TCA. The clean-up step included the use of SPE cartridges or PSA reagent and, in the case of the QuEChERS method, extraction salts such as MgSO _4_, Na _2_SO _4_ and NaCl, as well as adsorbents such as PSA, C18 and MgSO _4_. In general, the separation and detection method used to analyze antibiotic residues is high-performance liquid chromatography coupled to a triple quadrupole mass spectrometer (MS/MS) with Orbitrap or Q-ToF detectors. Validation of these methods in accordance with the provisions of Commission Implementing Regulation (EU) 2021/808 is essential to ensuring the reliability of the results obtained. The development of analytical strategies for the analysis of antibiotics in honey is crucial to allow faster and precise identification of these contaminants. In this sense, the advancement in emerging technologies and innovations in chromatography, mass spectrometry, and sensor technologies will enable more sensitive, specificity, and efficiency techniques, therefore ensuring more reliable results. Also, the changes and updates in policies and regulatory frameworks encompass a need to continuously improve analytical methods. These innovations and advancements in analytical chemistry as a result will safeguard consumer health regarding honey consumption and enhance honey as a food product with several functional properties and health benefits.

## Data Availability

No data are associated with this article.
