# Supplementary material for: Antibiotics in honey: a comprehensive review on occurrence and analytical methodologies
Source: Open Res Eur. 2024 Nov 8;4:125. Originally published 2024 Jun 24. [Version 2] doi: 10.12688/openreseurope.17664.2 (PMC11555330; doi:10.12688/openreseurope.17664.2)
Supplement: Peer review history

# Reviewer report: Reviewer response for version 2

**DOI:** 10.21956/openreseurope.20393.r45967

**Published:** 2024-11-13

**Authors:** Jelena ĆIRIĆ

**Affiliations:** 1 Institute of Meat Hygiene and Technology, Belgrade, Serbia

All the best!

Is the review written in accessible language?

Yes

Are all factual statements correct and adequately supported by citations?

Yes

Are the conclusions drawn appropriate in the context of the current research literature?

Yes

Is the topic of the review discussed comprehensively in the context of the current literature?

Yes

Reviewer Expertise:

Food safety; food quality, honey bee products, biotechnology, veterinary medicine

I confirm that I have read this submission and believe that I have an appropriate level of expertise to confirm that it is of an acceptable scientific standard.

# Reviewer report: Reviewer response for version 2

**DOI:** 10.21956/openreseurope.20393.r45966

**Published:** 2024-11-11

**Authors:** Juraj Majtan

**Affiliations:** 1 Institute of Molecular Biology, Slovak Academy of Sciences, Bratislava, Slovakia

The authors successfully addressed all my raised issues and suggestions/comments. The revised manuscript has been improved. I have no other comments to the authors.

Is the review written in accessible language?

Yes

Are all factual statements correct and adequately supported by citations?

Yes

Are the conclusions drawn appropriate in the context of the current research literature?

Yes

Is the topic of the review discussed comprehensively in the context of the current literature?

Yes

Reviewer Expertise:

apimedical science, functional foods. food chemistry

I confirm that I have read this submission and believe that I have an appropriate level of expertise to confirm that it is of an acceptable scientific standard.

# Reviewer report: Reviewer response for version 1

**DOI:** 10.21956/openreseurope.19089.r42564

**Published:** 2024-08-14

**Authors:** Juraj Majtan

**Affiliations:** 1 Institute of Molecular Biology, Slovak Academy of Sciences, Bratislava, Slovakia

Manuscript entitled “Antibiotics in honey: a comprehensive review on occurrence and analytical methodologies” focused on in-depth description of analytical methods evaluating the antibiotic contamination in honey. This is important topic to be discussed in the light of bees’ bacterial infections and the use of antibiotics to treat these infections in bee colonies.

 Overall, the manuscript is well designed and written. It provides an comprehensive up-to-date information. 

 Major comments.

1. According to EU legislation, the use of antibiotics in beekeeping is not allowed. The absence of MLRs for antibiotics’ residues means “zero tolerance”. Therefore, the detection limit of the used analytical methods is an important point.  The authors should emphasize this issue.
2. The chapter “Honey: nutritional composition & functional properties” should be rewritten and authors should highlight the health benefits of honey and try to answer following questions: Why it is important to consume honey regularly? What kind of diseases can be treated (if any)? Most importantly, it is needed to emphasize that honey functional properties and health benefits can be achieved with pure/uncontaminated honey without harsh technological processing or prolonged storage.
3. I suggest to add a chapter where future perspective (e.g. sensitive immunoenzyme assays, tac.)

Minor comments:

 Some statements seem to be misleading.

- Abstract should be shortened.
- catalase is not enzyme produced by bees. It is of pollen origin.
- “Honey has also been associated with various health benefits, such as antioxidant and antimicrobial activities” antioxidant and antimicrobial activities are not health benefits.
- Linking glucose oxidase with antioxidant properties is not current. Glucose oxidase enzyme is responsible for hydrogen peroxide generation.
- Nosema cerana is a not bacterial pathogen but a microsporidian parasite
- All used shortcuts in Tables (Table 5-7) need to be written in full as note under the tables.

Is the review written in accessible language?

Yes

Are all factual statements correct and adequately supported by citations?

Yes

Are the conclusions drawn appropriate in the context of the current research literature?

Yes

Is the topic of the review discussed comprehensively in the context of the current literature?

Yes

Reviewer Expertise:

apimedical science, functional foods. food chemistry

I confirm that I have read this submission and believe that I have an appropriate level of expertise to confirm that it is of an acceptable scientific standard, however I have significant reservations, as outlined above.

# Response

**Published:** 2024-10-29

**Authors:** Andreia Freitas

**Affiliations:** INIAV, Portugal

Manuscript entitled “Antibiotics in honey: a comprehensive review on occurrence and analytical methodologies” focused on in-depth description of analytical methods evaluating the antibiotic contamination in honey. This is important topic to be discussed in the light of bees’ bacterial infections and the use of antibiotics to treat these infections in bee colonies. Overall, the manuscript is well designed and written. It provides an comprehensive up-to-date information. 

**Authors’ response (A.R.)** **Major comments.**

1. According to EU legislation, the use of antibiotics in beekeeping is not allowed. The absence of MLRs for antibiotics’ residues means “zero tolerance”. Therefore, the detection limit of the used analytical methods is an important point.  The authors should emphasize this issue.

**A.R.:** *The authors agree with the comment of the reviewer and in that sense the following sentence was added in Page 9, Section “Regulatory frameworks”:* *“Given that veterinary medicines are not classified as MRL substances in honey, a “zero tolerance” approach is required. In terms of analytical strategies, that situation leads to the need to have methods with limits of detection as low as possible.*  

1. The chapter “Honey: nutritional composition & functional properties” should be rewritten and authors should highlight the health benefits of honey and try to answer following questions: Why it is important to consume honey regularly? What kind of diseases can be treated (if any)? Most importantly, it is needed to emphasize that honey functional properties and health benefits can be achieved with pure/uncontaminated honey without harsh technological processing or prolonged storage.

**A.R.:** *The authors appreciate the reviewer’s comment. The main focus of this review is the occurrence and analytical strategies due to contamination through antibiotics, and not on the nutritional evaluation of honey. Nonetheless, some insights were added to the chapter to give more emphasis on the nutritional importance of this food, namely:* *“Honey has also been associated with various health benefits and functional properties such as antioxidant and antimicrobial activities. It is crucial to underscore that the functional properties and health benefits associated with honey can be attained through the consumption of pure, uncontaminated honey, without the application of rigorous technological processing or prolonged storage.*  

1. I suggest to add a chapter where future perspective (e.g. sensitive immunoenzyme assays, tac.)

**A.R.:** *The authors agree that should be emphasized future perspectives on this matter and in that sense (and due to the articles’ length to be according to the journal guidelines) the following sentences/paragraphs were added:* ***Section “Methods for extraction and detection of antibiotics in honey”*** *The analysis of antibiotics in honey employs one of two principal methods, which are selected according to the objective of the determination. Multiclass analysis methods essentially employ confirmation methods, whereby an initial extraction step is conducted prior to the application of chromatographic methods coupled with mass spectrometry detection. The second method type pertains to screening tests employing microbiological and immunoenzymatic techniques, which are capable of detecting the presence of an antibiotic or a family of antibiotics at a specific level. These include microbiological tests and enzyme-linked immunosorbent assays, which are typically referenced. The principal advantages of this approach are its speed, simplicity, and low cost. Nevertheless, a frequently cited disadvantage is that it can produce false positive results (Guliy et al., 2022; Jakšić et al., 2018).* ***Section “Conclusions”*** *In the conclusions, the authors also added:* *The development of analytical strategies for the analysis of antibiotics in honey is crucial to allow faster and precise identification of these contaminants. In this sense, the advancement in emerging technologies and innovations in chromatography, mass spectrometry, and sensor technologies will enable more sensitive, specificity, and efficiency techniques, therefore ensuring more reliable results. Also, the changes and updates in policies and regulatory frameworks encompass a need to continuously improve analytical methods. These innovations and advancements in analytical chemistry as a result will safeguard consumer health regarding honey consumption and enhance honey as a food product with several functional properties and health benefits*.   

**Minor comments:** Some statements seem to be misleading.

- Abstract should be shortened.

**A.R.:** *Since the abstract is in line with the guidelines, with a max of 300 words acceptance, authors believe that is no need to reduce it.*

- catalase is not enzyme produced by bees. It is of pollen origin.

**A.R.:** *The authors appreciate the comment and have deleted the word “catalase”.*

- “Honey has also been associated with various health benefits, such as antioxidant and antimicrobial activities” antioxidant and antimicrobial activities are not health benefits.

**A.R.:** *The authors agree and have changed the sentence to “Honey has also been associated with various health benefits and functional properties such as antioxidant and antimicrobial activities.”*
**                                                                                                                       **

- Linking glucose oxidase with antioxidant properties is not current. Glucose oxidase enzyme is responsible for hydrogen peroxide generation.

**A.R.:** *The authors appreciate the comment and have deleted the word “glucose oxidase” from that sentence.*

- Nosema cerana is a not bacterial pathogen but a microsporidian parasite.

**A.R.:** *The authors have changed the title of that section to “Bee diseases” to encompass all the microorganims that affect bee health, and deleted “Bacterial”.*

- All used shortcuts in Tables (Table 5-7) need to be written in full as note under the tables.

**A.R.:** *The authors agree and have made the corresponding changes.*

**Is the topic of the review discussed comprehensively in the context of the current literature?** Yes

**Are all factual statements correct and adequately supported by citations?** Yes **Is the review written in accessible language?** Yes

**Are the conclusions drawn appropriate in the context of the current research literature?** Yes

# Reviewer report: Reviewer response for version 1

**DOI:** 10.21956/openreseurope.19089.r42561

**Published:** 2024-08-06

**Authors:** Jelena ĆIRIĆ

**Affiliations:** 1 Institute of Meat Hygiene and Technology, Belgrade, Serbia

Respected,

 After reading this manuscript, I realized that authors were examined the antibiotics in honey: a comprehensive review on occurrence and analytical methodologies. The issue that authors dealt with is well described in the paper, and this work complements previous knowledge and opens up the possibility for further research. Paper is technically and clearly written. Discussion is clearly written. The methodological details are explain well. Also, tables and figures are very clear presented.

 I believe that the manuscript is suitable for indexing in Journal. Certainly the results are important to scientific literature. My recommendation is to index the paper of the manuscript.

Is the review written in accessible language?

Yes

Are all factual statements correct and adequately supported by citations?

Yes

Are the conclusions drawn appropriate in the context of the current research literature?

Yes

Is the topic of the review discussed comprehensively in the context of the current literature?

Yes

Reviewer Expertise:

Food safety; food quality, honey bee products, biotechnology, veterinary medicine

I confirm that I have read this submission and believe that I have an appropriate level of expertise to confirm that it is of an acceptable scientific standard.
